# Draft Genome Sequence of Oenococcus kitaharae CRBO2176, Isolated from Homemade Water Kefir

**DOI:** 10.1128/mra.01072-22

**Published:** 2023-03-29

**Authors:** Yasma Barchi, Amel Chaib, Florencia Oviedo-Hernandez, Marion Decossas, Olivier Claisse, Claire Le Marrec

**Affiliations:** a University of Bordeaux, UMR 1366 INRAE, ISVV, Villenave d’Ornon, France; b University of Bordeaux, CBMN UMR 5248, Bordeaux INP, Pessac, France; University of Maryland School of Medicine

## Abstract

Here, we announce the draft genome sequence of an Oenococcus kitaharae strain isolated from homemade water kefir in Bordeaux, France. *O. kitaharae* CRBO2176 is deposited at the Biological Resources Center Oenology (CRBO) of the Institute of Vine and Wine Science (ISVV; Villenave d’Ornon, France).

## ANNOUNCEMENT

Lactic acid bacteria are important in various fermentation processes, for which some species are described as highly versatile, while others are adapted to specific niches (1). This is especially true in fermented beverages, which harbor distinct members of the genus *Oenococcus* (2). Wine and apple ciders are undoubtedly the favorite habitats of Oenococcus oeni species, and strains have progressively been domesticated to this complex environment (2, 3). A few strains of O. oeni (4), as well as members of the three sister species, namely, Oenococcus sicerae (5), O. alcoholitolerans (6), and O. kitaharae (7), have also been isolated from overripe fruits and beverages with low alcohol content, such as water kefir (8). Here, we report the draft genome sequence of an *O. kitaharae* isolate from water kefir to enrich the limited genomic resources of *Oenococcus* species and better understand the processes of adaptation to kefir.

A homemade kefir prepared from figs (Bordeaux, France) was plated onto red grape juice agar (9), and the plates were incubated at 25°C for 5 days under anaerobic conditions. Slow-growing isolates were purified three times by single-colony isolation. Among these, an isolate (CRBO2176) was selected, and colony PCR was performed using the universal 16S primers fD1 and rD1 (10). Sequence analysis of the amplicon showed 99.9% similarity to *O. kitaharae* DSM 17330 (GenBank accession number CM001398) based on the Ribosomal Database Project (RDP) Classifier algorithm (RDP trainset 18/release 11.5) (11). For whole-genome sequencing, the strain was statically cultivated for 72 h at 25°C in MRS broth (pH 6). Genomic DNA was extracted using the Promega DNA Wizard kit (2) and submitted to the Genome-Transcriptome Facility of Bordeaux for library preparation (QIAseq FX DNA; Qiagen, Courtaboeuf, France) and whole-genome sequencing (Illumina MiSeq v3), producing 2 × 254-bp paired-end reads.

Default parameters were used for all software unless otherwise specified. A total of 445,075 raw read pairs were obtained, trimmed using Trimmomatic v0.39 (12), and assembled using SKESA v2.4.0 (13), generating 7 contigs with a genome length of 1,754,187 bp and 66-fold coverage (Table 1). Plasmids and prophages were absent from CRBO2176 according to PlasmidFinder v2.1.1 (14) and PHASTER (Web service; accessed August 2022) (15). The NCBI Prokaryotic Genome Annotation Pipeline (PGAP) v6.2 (16) was used to predict 1,771 genes, with 1,721 coding DNA sequences, 44 tRNA genes, and 3 rRNA genes.

**TABLE 1:** Characteristics of the kefir isolate and genome assembly in this study

Parameter	Finding
No. of contigs	7
Largest contig (bp)	647,507
Total length (bp)	1,754,187
*N*_50_ (bp)	578,835
*N*_75_ (bp)	411,412
*L*_50_	2
*L*_75_	3
GC content (%)	42.77

Like other members of the genus *Oenococcus*, strain CRBO2176 can reliably be distinguished from all other *Leuconostocaceae* and *Lactobacillaceae* species by the presence of conserved signature indels (CSIs) in 13 proteins: DNA-directed RNA polymerase subunit beta, two different CSIs in translocase subunit SecY, 50S ribosomal protein L13, molecular chaperone DnaK, riboflavin kinase, Sua5/YciO/YrdC/YwlC family protein, RluA family pseudouridine synthase, amino acid permease, TatD family hydrolase, YidC/OxaI family membrane protein insertase, class I *S*-adenosylmethionine (SAM)-dependent RNA methyltransferase, and GTPase HflX (17).

**FIG 1:**
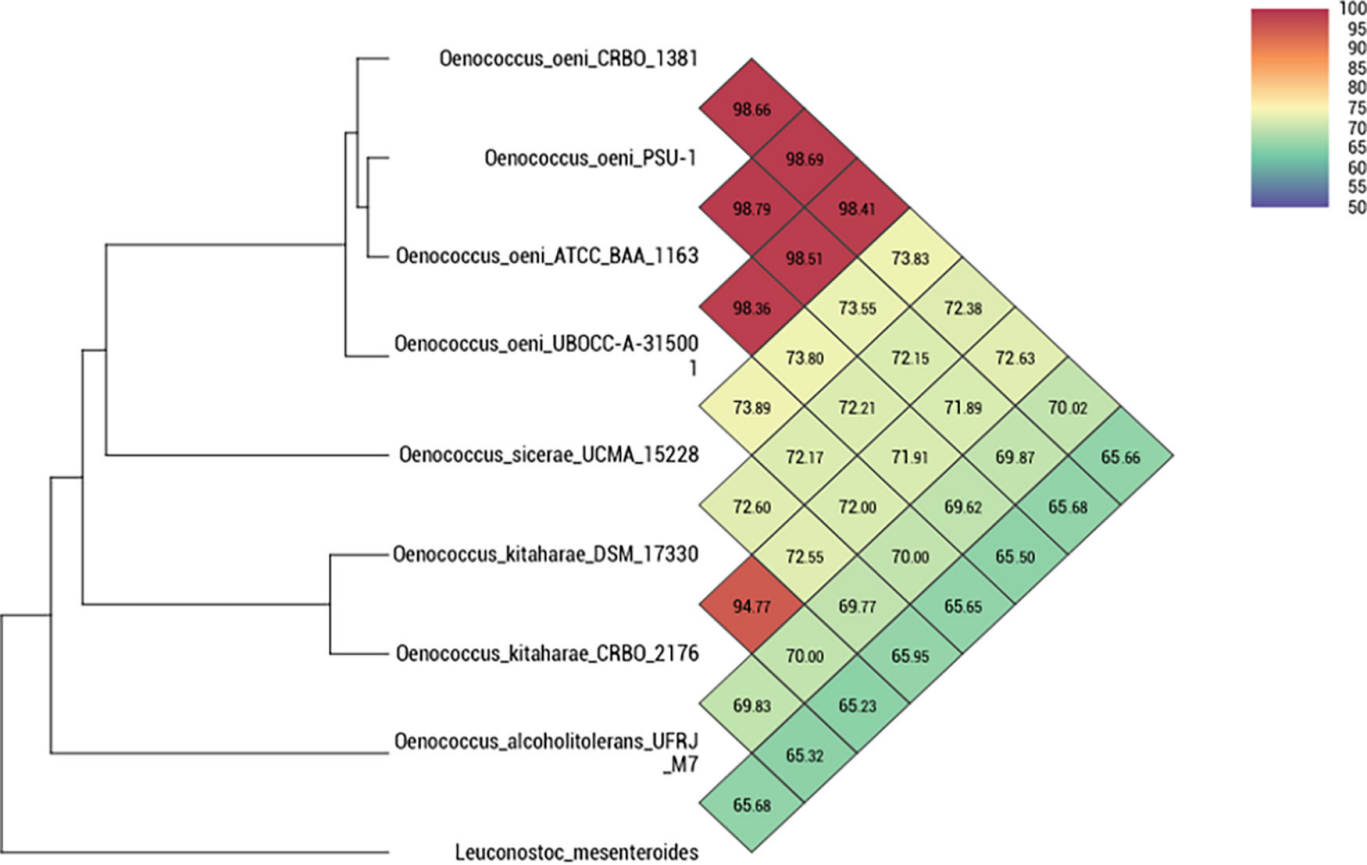
Heatmap showing the average nucleotide identity by orthology (OrthoANI) between strain CRBO2176 and all the type strains of the genus *Oenococcus*, calculated using OAT software, with Leuconostoc mesenteroides ATCC 8293 (GenBank accession number GCA_000014445.1) used as the outgroup.

The draft genome showed a Mash (18) (MinHash v1.1) distance of 0.0333 and an average nucleotide identity of 94.77% (calculated using OrthoANI v1.40 [19]) with the type strain DSM 17330 (GenBank accession number NZ_CM001398), isolated from shochu (Fig. 1).

### Data availability.

The 16S rRNA gene sequence, genome sequence, and raw sequencing reads for CRBO2176 have been deposited at GenBank under accession numbers OP985037 and JANJQP000000000, BioProject accession number PRJNA865506, BioSample accession number SAMN30114423, and SRA accession number SRR21928607.

## References

[1] Tamang JP, Cotter PD, Endo A, Han NS, Kort R, Liu SQ, Mayo B, Westerik N, Hutkins R. 2020. Fermented foods in a global age: east meets west. Compr Rev Food Sci Food Saf 19:184–217. doi:10.1111/1541-4337.12520. 33319517

[2] Campbell-Sills H, El Khoury M, Favier M, Romano A, Biasioli F, Spano G, Sherman DJ, Bouchez O, Coton E, Coton M, Okada S, Tanaka N, Dols-Lafargue M, Lucas PM. 2015. Phylogenomic analysis of Oenococcus oeni reveals specific domestication of strains to cider and wines. Genome Biol Evol 7:1506–1518. doi:10.1093/gbe/evv084. 25977455 PMC4494047

[3] Lorentzen MPG, Lucas PM. 2019. Distribution of Oenococcus oeni populations in natural habitats. Appl Microbiol Biotechnol 103:2937–2945. doi:10.1007/s00253-019-09689-z. 30788540 PMC6447504

[4] Zanirati DF, Abatemarco M, de Cicco Sandes SH, Nicoli JR, Nunes AC, Neumann E. 2015. Selection of lactic acid bacteria from Brazilian kefir grains for potential use as starter or probiotic cultures. Anaerobe 32:70–76. doi:10.1016/j.anaerobe.2014.12.007. 25542841

[5] Verce M, De Vuyst L, Weckx S. 2020. The metagenome-assembled genome of Candidatus Oenococcus aquikefiri from water kefir represents the species Oenococcus sicerae. Food Microbiol 88:103402. doi:10.1016/j.fm.2019.103402. 31997765

[6] Badotti F, Moreira APB, Tonon LAC, de Lucena BTL, de Cássia O Gomes F, Kruger R, Thompson CC, de Morais MA, Jr, Rosa CA, Thompson FL. 2014. Oenococcus alcoholitolerans sp. nov., a lactic acid bacteria isolated from cachaça and ethanol fermentation processes. Antonie Van Leeuwenhoek 106:1259–1267. doi:10.1007/s10482-014-0296-z. 25315101

[7] Endo A, Okada S. 2006. Oenococcus kitaharae sp. nov., a non-acidophilic and non-malolactic-fermenting Oenococcus isolated from a composting distilled shochu residue. Int J Syst Evol Microbiol 56:2345–2348. doi:10.1099/ijs.0.64288-0. 17012559

[8] Lynch KM, Wilkinson S, Daenen L, Arendt EK. 2021. An update on water kefir: microbiology, composition and production. Int J Food Microbiol 345:109128. doi:10.1016/j.ijfoodmicro.2021.109128. 33751986

[9] Chaïb A, Philippe C, Jaomanjaka F, Claisse O, Jourdes M, Lucas P, Cluzet S, Le Marrec C. 2019. Lysogeny in the lactic acid bacterium Oenococcus oeni is responsible for modified colony morphology on red grape juice agar. Appl Environ Microbiol 85:e00997-19. doi:10.1128/AEM.00997-19. 31375489 PMC6752011

[10] Weisburg WG, Barns SM, Pelletier DA, Lane DJ. 1991. 16S ribosomal DNA amplification for phylogenetic study. J Bacteriol 173:697–703. doi:10.1128/jb.173.2.697-703.1991. 1987160 PMC207061

[11] Cole JR, Wang Q, Fish JA, Chai B, McGarrell DM, Sun Y, Brown CT, Porras-Alfaro A, Kuske CR, Tiedje JM. 2014. Ribosomal Database Project: data and tools for high throughput rRNA analysis. Nucleic Acids Res 42:D633–D642. doi:10.1093/nar/gkt1244. 24288368 PMC3965039

[12] Bolger AM, Lohse M, Usadel B. 2014. Trimmomatic: a flexible trimmer for Illumina sequence data. Bioinformatics 30:2114–2120. doi:10.1093/bioinformatics/btu170. 24695404 PMC4103590

[13] Souvorov A, Agarwala R, Lipman DJ. 2018. SKESA: strategic k-mer extension for scrupulous assemblies. Genome Biol 19:153. doi:10.1186/s13059-018-1540-z. 30286803 PMC6172800

[14] Carattoli A, Zankari E, García-Fernández A, Voldby Larsen M, Lund O, Villa L, Møller Aarestrup F, Hasman H. 2014. In silico detection and typing of plasmids using PlasmidFinder and plasmid multilocus sequence typing. Antimicrob Agents Chemother 58:3895–3903. doi:10.1128/AAC.02412-14. 24777092 PMC4068535

[15] Arndt D, Grant JR, Marcu A, Sajed T, Pon A, Liang Y, Wishart DS. 2016. PHASTER: a better, faster version of the PHAST phage search tool. Nucleic Acids Res 44:W16–W21. doi:10.1093/nar/gkw387. 27141966 PMC4987931

[16] Tatusova T, DiCuccio M, Badretdin A, Chetvernin V, Nawrocki EP, Zaslavsky L, Lomsadze A, Pruitt KD, Borodovsky M, Ostell J. 2016. NCBI Prokaryotic Genome Annotation Pipeline. Nucleic Acids Res 44:6614–6624. doi:10.1093/nar/gkw569. 27342282 PMC5001611

[17] Bello S, Rudra B, Gupta RS. 2022. Phylogenomic and comparative genomic analyses of Leuconostocaceae species: identification of molecular signatures specific for the genera Leuconostoc, Fructobacillus and Oenococcus and proposal for a novel genus Periweissella gen. nov. Int J Syst Evol Microbiol 72:005284. doi:10.1099/ijsem.0.005284. 35320068 PMC9558574

[18] Ondov BD, Treangen TJ, Melsted P, Mallonee AB, Bergman NH, Koren S, Phillippy AM. 2016. Mash: fast genome and metagenome distance estimation using MinHash. Genome Biol 17:132. doi:10.1186/s13059-016-0997-x. 27323842 PMC4915045

[19] Lee I, Kim YO, Park S-C, Chun J. 2016. OrthoANI: an improved algorithm and software for calculating average nucleotide identity. Int J Syst Evol Microbiol 66:1100–1103. doi:10.1099/ijsem.0.000760. 26585518

